# Hyperinflammation and immunomodulation in influenza, SARS-CoV-2 and syncytial respiratory virus infection in adults: from innate immune responses and biomarkers to clinical and therapeutic implications

**DOI:** 10.3389/fmicb.2026.1916526

**Published:** 2026-09-29

**Authors:** Jesus Alonso-Carrillo, Marta Obra, Mario Cereza, María Ruiz-Ruigomez, Carlos Lumbreras, Antonio Lalueza

**Affiliations:** 1 Department of Internal Medicine, Hospital Universitario 12 de Octubre, Madrid, Spain; 2 Department of Medicine, School of Medicine, Complutense University of Madrid, Madrid, Spain; 3 Research Institute Hospital 12 de Octubre (i+12), Madrid, Spain; 4 Centro de Investigaciones Biomédicas en Red de Enfermedades Infecciosas (CIBERINFEC), Madrid, Spain; 5 Unit of Infectious Diseases, Hospital Universitario “12 de Octubre, ” Madrid, Spain

**Keywords:** COVID-19, cytokine, IL-1, IL-6, immunomodulation, influenza, JAK inhibitors, respiratory syncytial virus

## Abstract

Influenza, SARS-CoV2-2 and syncytial respiratory virus infection in adults are major causes of morbidity and mortality. However, the pathophysiological mechanisms underlying these infections remain incompletely understood. Disease severity results from an interaction between viral factors and host responses. Lung damage and respiratory failure are produced by hyperinflammation. Understanding these mechanisms is necessary to improve risk stratification and may guide therapeutic decisions. C- reactive protein (CRP), IL-6, ferritin, and ApoH are relevant biomarkers which can identify inflammatory phenotypes. Antivirals and supportive care are the main approaches for these viral infections. From a therapeutic perspective, broad immunosuppression is unlikely to be optimal, and strategies should focus on selectively modulating harmful inflammation while preserving antiviral immunity. Nevertheless, there is scarce evidence of the best choice of treatment based on the patient’s clinical profile and further studies are needed.

## 1 Introduction

After the COVID-19 pandemic, respiratory viral infections such as influenza, severe acute respiratory syndrome coronavirus 2 (SARS-CoV-2), and respiratory syncytial virus (RSV) have brought attention back to respiratory viral infections (Li L. et al., 2025; Zhao P. et al., 2024). Nowadays, these infections remain main cause of hospitalization and mortality, particularly among older adults, individuals with comorbidities, and immunocompromised patients (Silk et al., 2026). Among the comorbidities associated with the highest risk of hospital admission due to respiratory infection, diabetes, chronic obstructive pulmonary disease and heart failure are the most significant (Prasad et al., 2021; Dalfardi et al., 2026). The progressive aging of the population is further increasing the burden of these infections on healthcare systems (Díez-Domingo et al., 2014). Sporadic reports from some advanced countries like the United States of America have reported the incidence among individuals aged ≥ 75 years old which is the highest, 932.6 cases per 100,000 inhabitants, followed by influenza (598.8 cases per 100,000 inhabitants) and respiratory syncytial virus (426.9 cases per 100,000 inhabitants) (Osei-Yeboah et al., 2023; Silk et al., 2026). A cross-sectional study conducted in the United States between 1999 and 2018 reported that adults aged ≥ 65 years had mortality rates of 14.7 deaths per 100,000 population for RSV and 20.5 deaths per 100,000 population for influenza (Hansen et al., 2022). Moreover, economic burden of RSV hospitalizations was $1.3 billion in adults (Grace et al., 2023) and $2.2 billion in influenza hospitalizations (Bolek et al., 2022).

The clinical manifestations of viral respiratory infections are diverse and include nasal congestion, rhinorrhea, and lower respiratory tract infection. These infections can lead to a variety of severe complications, such as pneumonia, hypoxemic respiratory failure, and the necessity for hospitalization. In the most severe cases of respiratory failure, there may be progressive deterioration, which may require advanced respiratory support, and can ultimately result in multiorgan dysfunction and death (Lee et al., 2013; Wildenbeest et al., 2024).

In recent years, preventive strategies have developed. Vaccination remains a mainstay of protection against influenza and SARS-CoV-2 (Scott et al., 2025), while recent studies have led to the approval of the first active immunization strategies against RSV. These vaccines aim to protect older adults and pregnant women to confer protection to infants (Falsey et al., 2023; Walsh et al., 2023). However, therapeutic options remain limited and focus on supportive care and some antiviral options. For influenza, oseltamivir is well-established and widely used (Nicholson et al., 2000). Whereas treatment strategies for COVID-19 combine antiviral and anti-inflammatory approaches according to disease phase and severity (The Recovery Collaborative Group, 2021). For RSV, management in adults remains largely supportive and there is no specific antiviral therapy, except for severely immunocompromised patients. These limitations highlight the need the need to understand the mechanisms that drive progression from uncomplicated infection to severe respiratory disease and lung injury (Von Lilienfeld-Toal et al., 2026).

Viral replication induces direct cytopathic injury to infected epithelial cells. This includes apoptosis, metabolic exhaustion, and structural disruption. Nevertheless, while direct viral injury contributes to early tissue damage, the severity of pulmonary dysfunction in severe respiratory viral infections is strongly driven by host immune responses (Newton et al., 2016).

The mechanisms underlying disease severity and clinical heterogeneity in adult virus infections remain a largely unknown field. In particular, the balance between viral factors and host immune responses, as well as the identification of reliable biomarkers should be mentioned as a major clinical challenge.

Understanding these mechanisms is essential to improve risk assessment, guide treatments, and optimize patient outcomes. In this narrative review, our aim is to summarize an up to date overview of the interaction of the main viral respiratory infections (influenza, SARS-CoV-2, and RSV) and the early innate immune response in adults, as well as its clinical and therapeutic implications.

## 2 Methods

We have designed a narrative review. A literature search was performed in the MEDLINE (PubMed), EMBASE and Cochrane Library databases without date restrictions. The search strategy included Medical Subject Headings (MeSH) and free-text terms. The MeSH words used were: “Influenza, Human,” “COVID-19,” “SARS-CoV-2,” “Respiratory Syncytial Virus Infections,” “Immunity, Innate,” “Inflammation,” “Cytokines,” “Interleukin-6,” “Interleukine-1,” “Interferons,” “Biomarkers,” “Ferritins,” “Anti-Inflammatory Agents,” “Janus Kinase Inhibitors,” “Respiratory Distress Syndrome,” together with the free-text terms “hyperinflammation,” “respiratory viral infections,” “innate immunity,” “biomarkers,” “immunomodulatory therapies,” “cytokine storm,” “immune dysregulation,” “immunomodulation,” “NETosis,” “PTX3,” and “ApoH,” with a particular focus on influenza, SARS-CoV-2, and RSV.

Only articles published in English were included. Original research articles, randomized controlled trials, observational studies, systematic reviews, meta-analyses, and relevant clinical practice guidelines were eligible for inclusion. References were selected based on their scientific quality, clinical relevance, and contribution to the objectives of this review.

The literature was independently reviewed by three infectious disease specialists who selected the most relevant studies according to its quality, clinical relevance and contribution to the objectives of the review. The discrepancies were resolved through consensus among the three reviewers. Given the narrative nature of this work, no formal systematic review methodology, risk of bias assessment, or quantitative evidence synthesis was performed. This review primarily focuses on respiratory viral infections in adults. However, chosen pediatric studies were included, particularly in the context of RSV infection if no data was available for adults.

## 3 Review

### 3.1 Pathophysiology of the immune response to viral infection

The pathophysiology of the immune response is a complex process in which disease severity is determined not only by viral cytopathic effect, but also by the host’s immune response. There is a critical interaction between innate and adaptive responses that ultimately shapes the balance between effective viral clearance and immune-mediated tissue injury (Newton et al., 2016).

#### 3.1.1 Mechanical and chemical barrier defense

The respiratory tract constitutes the first line of defense through mucociliary clearance and chemical barriers. Ciliated epithelial cells trap and remove pathogens by secreting antimicrobial factors, such as surfactant, complement, mucins and antimicrobial peptides, which provides an active resource against infections (Gambadauro et al., 2024).

#### 3.1.2 Innate immune response

The innate immune response inhibits viral replication, protecting host cells, and promoting the elimination of infected cells (Mueller and Rouse, 2008). Innate immune activation begins with the recognition of conserved and unique molecular structures of pathogens, known as pathogen-associated molecular patterns (PAMPs) (Mogensen, 2009). These structures are detected by host sensors called pattern recognition receptors (PRRs), which are found in epithelial and immune cells. Based on protein domain homology, five receptor families have been identified, each activated by their respective ligands in different subcellular compartments (Li and Wu, 2021; Table 1).

**TABLE 1:** Pattern recognition receptors (PRRs) involved in antiviral innate immune responses: families, ligands, and biological effect.

PRR family	Main receptors	Cellular location	Viral ligand recognized	Principal biological outcome
RIG-I-like receptors (RLRs)	RIG-I, MDA5, LGP2	Cytoplasm	Viral RNA	Type I interferon production; proinflammatory cytokines
Toll-like receptors (TLRs)	TLR3, TLR7, TLR8, TLR9, TLR10	Endosomes	Viral RNA/DNA	Type I IFN induction; inflammatory cytokine production
C-type lectin receptors (CLRs)	DC-SIGN, mannose receptor, MDL-1	Cell surface/Endosomes	Glycosylated viral proteins	Cytokine production; modulation of innate immunity
NOD-like receptors (NLRs)	NLRP3	Cytoplasm	Cellular stress/viral RNA-associated signals	IL-1β and IL-18 maturation; pyroptosis
STING pathway	STING	Endoplasmic reticulum	Cytosolic DNA/DNA-derived signals	Type I interferon production

PRR, pattern recognition receptor; RLRs, RIG-I-like receptors; RIG-I, retinoic acid-inducible gene I; MDA5, melanoma differentiation-associated protein 5; LGP2, laboratory of genetics and physiology 2; TLRs, Toll-like receptors; CLRs, C-type lectin receptors; NLRs, NOD-like receptors; NLRP3, NOD-, LRR-, and pyrin domain-containing protein 3; STING, stimulator of interferon genes; RNA, ribonucleic acid; DNA, deoxyribonucleic acid; IFN, interferon; IL, interleukin; LRR, leucine-rich repeat.

These signals trigger the release of type III interferons, IFN-λ, and type I IFNs, IFN-α/β. Type III IFNs act locally and are responsible for controlling early viral spread producing low inflammation. Type I IFNs exert a more potent, systemic antiviral response that may also contribute to organ damage (Gambadauro et al., 2024; Newton et al., 2016).

Firstly, type I IFNs activate the JAK–STAT signaling pathway, specifically, the receptor-associated kinases JAK1 and TYK2 are activated. Secondly, there is a phosphorylation of tyrosine residues in the intracellular domain of IFNAR and a recruitment and activation of STAT transcription factors (Abbas et al., 2024). These form the ISGF3 complex, which drives the transcription of interferon-stimulated genes and establishes an antiviral state in infected tissues (Abbas et al., 2024; Lamers and Haagmans, 2022; Newton et al., 2016; Piperakis et al., 2024). Finally, type I IFNs support antigen presentation to CD8+ T lymphocytes and increase the cytotoxic activity of natural killer (NK) cells (Abbas et al., 2024; Gambadauro et al., 2024).

Natural killer cells play a main role in innate immunity. NK cell activation is driven by IL-12, IL-15, IL-18, and type I IFNs. After recognizing virus infected cells, NK cells can destroy them through perforin and granzyme cytolysis, and produce IFN-y (Gambadauro et al., 2024; Piersma and Brizić, 2022). Moreover, they can produce cytokines and chemokines which attract other immune cells, promote direct antiviral effects or polarize the immune response (Piersma and Brizić, 2022). Invariant natural killer T cells are a subset of T cells that should be noticed. In addition to conventional NK cells, they contribute to antiviral immunity linking innate and adaptive response (Jeong et al., 2023). They enhance dendritic cell maturation, T and B cell response and Th1, Th2, or Th17 polarization.

Neutrophils are also recruited to the airways within hours of viral infection. This recruitment is driven by chemokines such as CXCL1, CXCL2, and CXCL8 after viral recognition by TLR3, TLR7, TLR8, TLR4, and RIG-I. They have different protective antimicrobial functions (Johansson and Kirsebom, 2021; Silva et al., 2023). Neutrophils opsonize viral particles and release antimicrobial granules that inhibit viral replication. In addition, neutrophil extracellular traps (NETs) are produced and they neutralize viral particles. Reactive oxygen species are also released (Muraro et al., 2018; Johansson and Kirsebom, 2021).

While IFNs are produced, proinflammatory cytokines such as interleukin-6 (IL-6), chemokines, and tumor necrosis factor-alpha (TNF-α) play an important role in innate immunity. They are mediators that coordinate the host inflammatory response to respiratory viral infections (Mantovani and Garlanda, 2023; Oseghale et al., 2022). These mediators are essential for facilitating the transition from early innate to adaptive immunity, whereas excessive cytokine production is frequently associated with severe tissue damage and clinical worsening (Newton et al., 2016).

On the one hand, IL-6 promotes the transition from innate to adaptive immunity by recruiting and differentiating monocytes and T cells and this cytokine induces the production of acute-phase proteins (Newton et al., 2016). Each chemokine plays a different role regulating leukocyte migration to the site of infection: CXCL8 recruits neutrophils; CCL2 promotes inflammatory monocytes and macrophages, and CXCL10 and CCL5 attract T cells, NK cells, and dendritic cells (Gambadauro et al., 2024). TNF-α increases inflammation, inhibits viral replication, promotes cytotoxic activity, and activates endothelial cells (Bennett et al., 2020). Moreover, elevated cytokines and chemokines drive the recruitment of inflammatory immune cells (monocytes, macrophages, dendritic cells, and neutrophils) to the site of infection (Li and Xu, 2022).

Acute-phase proteins are key components of innate immunity produced by the liver in response to the interleukins mentioned (IL-6 and IL-1). They are responsible for enhancing antimicrobial resistance and tissue repair, acting as primitive antibody-like molecules (Mantovani and Garlanda, 2023). These proteins enhance local and systemic immunity and trigger adaptive immune responses (Mantovani and Garlanda, 2023).

#### 3.1.3 Pulmonary surfactant and collections

Pulmonary surfactant, secreted by alveolar type II and club cells, reduces surface tension and contributes to innate antiviral defense. The hydrophobic SP-B and SP-C determine its biophysical properties, while hydrophilic SP-A and SP-D act as soluble pattern recognition receptors (Nayak et al., 2012).

SP-A and SP-D bind viral glycoprotein glycans, promoting aggregation, neutralization, opsonization, and debris clearance while modulating inflammation (Nayak et al., 2012). In influenza, SP-D inhibits hemagglutination (Hartshorn et al., 2000). SP-A/SP-D-deficient mice show increased replication and lung inflammation (LeVine et al., 2001) and polymorphic variants have been associated with respiratory disease (Herrera-Ramos et al., 2014). In SARS-CoV-2, SP-D binds spike S1/RBD, blocking ACE2 binding. Furthermore, a recombinant fragment of human SP-D reduces viral infectivity and replication *in vitro* (Madan et al., 2021). In RSV, SP-A opsonizes the virus and enhances its phagocytosis, and polymorphic variants have been consistently associated with severe bronchiolitis in infants (LeVine et al., 2004; Löfgren et al., 2002; Lahti et al., 2002).

#### 3.1.4 Adaptive immune response

Adaptive immunity is essential for eliminating viral infections and establishing long-lasting immunological memory (Mettelman et al., 2022). Three main cell populations are involved: B cells, CD4+ T helper cells (including Th1, Th2, Th17, regulatory T cells, and T follicular helper cells) and CD8+ cytotoxic T lymphocytes (Newton et al., 2016; Gambadauro et al., 2024).

The adaptive response is initiated when dendritic cells capture viral antigens in the respiratory mucosa and present them to naïve T cells, leading to their expansion and differentiation (24). CD8+ cytotoxic T lymphocytes eliminate infected cells by releasing perforin and granzyme B. However, excessive or dysregulated cytotoxic responses may contribute to tissue damage and airway dysfunction (Georgakopoulou and Pitiriga, 2025).

CD4+ T helper cells coordinate the broader immune response. On one side, Th1 cells support viral clearance by activating macrophages and producing IFN-γ and IL-2. In addition, Th2 cells drive type 2 inflammation and support humoral immunity through IL-4, IL-5, IL-9, and IL-13; and Th17 cells promote neutrophilic infiltration in the lung (Gambadauro et al., 2024). Regulatory T cells limit effector responses. RSV infection often promotes Th2 and Th17 polarization, which has been linked to eosinophilic inflammation, mucus hypersecretion, neutrophil recruitment, and epithelial barrier dysfunction (Newton et al., 2016; Georgakopoulou and Pitiriga, 2025).

B cells, through interactions with T follicular helper cells, differentiate into plasma cells that produce virus-specific antibodies, which contribute to viral clearance through neutralization, opsonization, and complement activation (Newton et al., 2016). In influenza, neutralizing antibodies are directed mainly against haemagglutinin and neuraminidase. Antigenic drift of these glycoproteins accounts for the need for annual vaccination, whereas cross-reactive CD8+ T cells recognizing conserved internal proteins may provide broader protection against severe disease.

In SARS-CoV-2 infection, humoral responses target the spike protein, and severe disease is characterized by marked CD4+ and CD8+ lymphopenia and features of T-cell exhaustion. In RSV, antibodies particularly target the prefusion F glycoprotein, thereby blocking viral entry (Georgakopoulou and Pitiriga, 2025), an antigen that forms the basis of the recently approved RSV vaccines for older adults. However, RSV has developed multiple mechanisms to evade humoral immunity. Those include the secretion of a soluble form of the G glycoprotein that neutralizes antibodies, and the limitation of durable immunological memory through inhibition of germinal center reactions and rapid waning of mucosal IgA responses (Teng et al., 2001). Consequently, reinfections are common throughout life (Georgakopoulou and Pitiriga, 2025).

Type I (IgE-mediated) hypersensitivity represents an additional link between respiratory viral infection and inflammatory airway injury. Following Th2 polarization, IL-4 and IL-13 promote B cell class switching to IgE, which binds the high-affinity receptor FcεRI on mast cells and basophils (Shibata et al., 2023). This triggers degranulation, with release of histamine, tryptase, leukotrienes, and prostaglandins, leading to bronchoconstriction, mucus hypersecretion and eosinophil recruitment. Beyond this classic allergic pathway, IgE may also influence antiviral immune responses while reflecting an underlying atopic predisposition. The clinical relevance of this axis has been best characterized in infants. In a multicenter prospective cohort of infants hospitalized for bronchiolitis, IgE-defined phenotypes were associated with differing risks of subsequent asthma development and with innate immune profiles (Shibata et al., 2023).

#### 3.1.5 Transition from innate to adaptive immune response

The immune response to respiratory viral infections relies on bidirectional coordination between the innate and adaptive immune systems, which are interconnected through several key mechanisms.

Dendritic cells (DCs) constitute the principal bridge between innate and adaptive immunity, as they produce several cytokines and migrate to secondary lymphoid organs, where they present viral antigens to naïve T lymphocytes, initiating the adaptive immune response.

Type I and type III interferons, IL-1, IL-6, recruit inflammatory cells and promote the maturation of antigen-presenting cells. This inflammatory environment also activates other innate immune cells, including natural killer cells and polymorphonuclear neutrophils which contribute to viral clearance (Soto et al., 2020).

A continuous bidirectional interaction exists between the innate and the adaptive immunity. Activated T-cells produce cytokines that regulate the activity of macrophages and neutrophils, whereas antibodies promote innate mechanisms such as opsonization and phagocytosis (Asha et al., 2021).

### 3.2 Resolution and return to homeostasis

Resolution of the immune response is a highly coordinated process. The host must transit from active resistance to tolerance and tissue repair. This step is essential to prevent excessive tissue damage and the development of chronic inflammatory states (Newton et al., 2016). During resolution, proinflammatory cells and chemokines are progressively replaced by anti-inflammatory mediators. The most relevant are interleukin-10 (IL-10) and transforming growth factor-beta (Yunis et al., 2023), produced by regulatory T cells (Tregs). They maintain homeostasis during infection and regulate effector responses promoting tissue repair through different mediators such as amphiregulin (Mettelman et al., 2022).

Additional regulatory mechanisms have been described. For instance, apolipoprotein H (ApoH) suppresses TLR4/NF-κB signaling pathway in macrophages. ApoH reduces the production of proinflammatory cytokines and promotes IL-10 expression (Yu Z. et al., 2024). Moreover, adenosine signaling also contributes to immune resolution. The return to immune quiescence is driven by inhibiting phagocyte activity and promoting the induction and survival of Tregs (Bennett et al., 2020). Restoration of tissue integrity depends on the efficient clearance of cellular debris by macrophages through efferocytosis. As this occurs, macrophages shift from a proinflammatory M1 phenotype, responsible for pathogen clearance, to an anti-inflammatory M2 phenotype that drives tissue repair and limits further immune recruitment (Yu Z. et al., 2024).

An inappropriate resolution of the inflammatory response may lead to persistent immune activation. This contributes to tissue damage and poor clinical outcomes. A pattern described across multiple respiratory viral infections, including RSV, influenza, and SARS-CoV-2.

### 3.3 Immune dysregulation and hyperinflammation

#### 3.3.1 Cytokine storm

The immune system is designed to recognize pathogens, respond proportionally, and ultimately return to homeostasis. While the immune system plays a vital role in the clearance of viruses, as it has been previously described, dysregulated immune activation can lead to pulmonary injury (Georgakopoulou and Pitiriga, 2025). Cytokine storm, is characterized by elevated circulating cytokine levels, systemic inflammatory symptoms, and secondary organ dysfunction exceeding what would be expected from a normal immune response to a pathogen, or by cytokine-driven dysfunction in the absence of infection (Fajgenbaum and June, 2020; Karki et al., 2026). However, hyperinflammation is a broad clinical concept that includes any inflammatory response that is deleterious to the host, regardless of cause (Keskitalo et al., 2025). Pathogenesis of cytokine storm is summarized in Figure 1.

**FIGURE 1:**
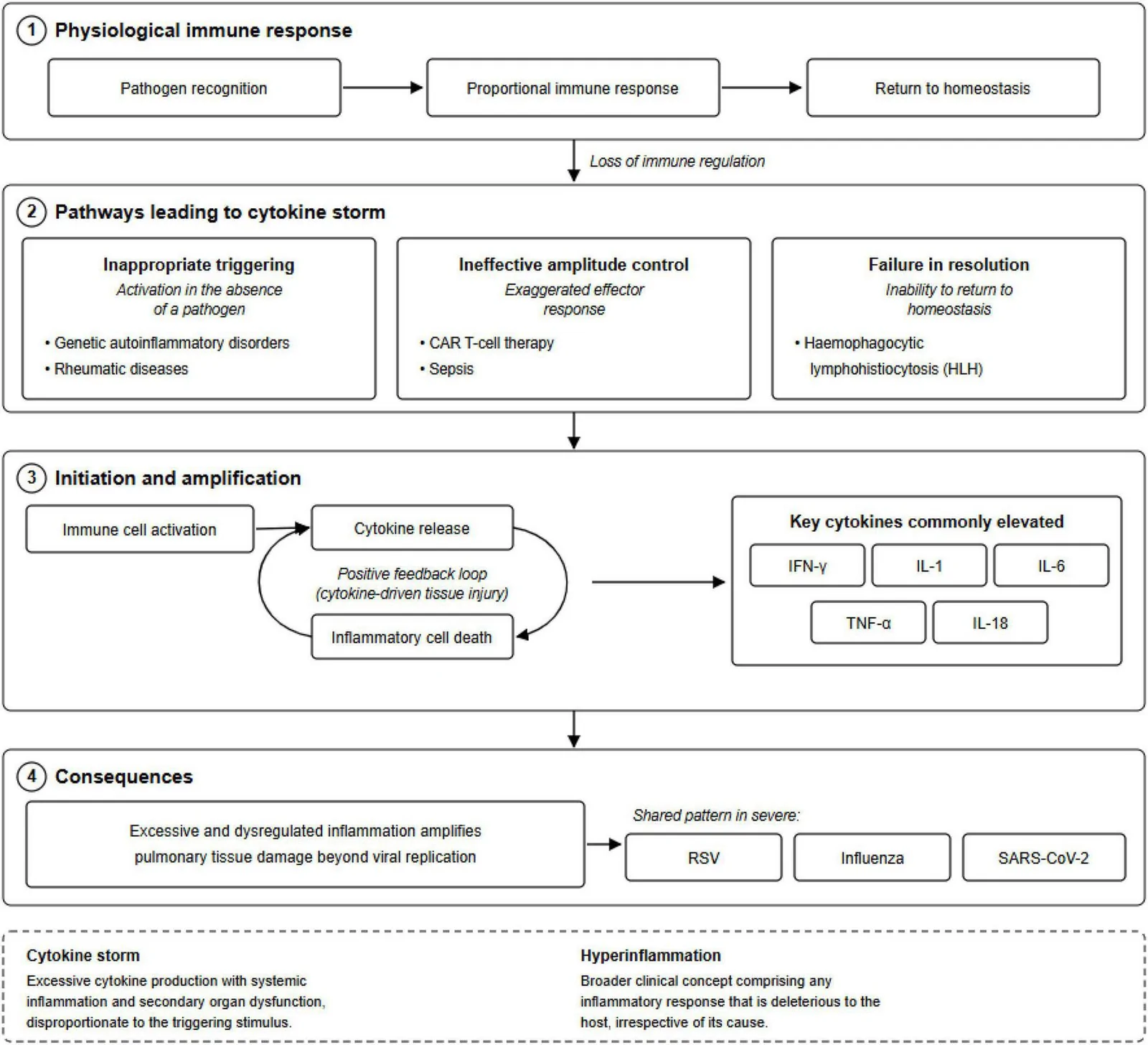
Pathogenesis of cytokine storm.

Cytokine storm may develop through several pathways. These include inappropriate triggering (activation in the absence of a pathogen, such as genetic autoinflammatory disorders or rheumatic diseases), ineffective amplitude control (exaggerated effector response, such as in CAR T cell therapy or sepsis), and failure in resolution (inability to return to homeostasis, such as hemophagocytic lymphocytosis) (Chatham, 2024; Fajgenbaum and June, 2020; Karki et al., 2026).

The process begins with the immune cell activation and is amplified by a positive feedback between cytokine release and inflammatory cell death which further drives cytokine production and tissue injury (Karki et al., 2026).

Cytokine storms involve a complex and interconnected network of immune cells, signaling pathways, and soluble mediators. Among the cytokines most commonly elevated it should be highlighted IFN-γ, IL-1, IL-6, TNFα, and IL-18 (Nie et al., 2025). Hyperinflammatory is a major contributor to disease severity in RSV, influenza, and SARS-CoV-2 infection (Ferrarese et al., 2026). An excessive and dysregulated immune response amplifies pulmonary tissue damage beyond viral replication.

#### 3.3.2 NETosis and tissue injury

NETosis is a specialized neutrophil response characterized by the release of neutrophil extracellular traps (NETs). These structures are made up of decondensed chromatin associated with antimicrobial proteins (neutrophil elastase and myeloperoxidase). Together, they trap and neutralize pathogens (Sebina and Phipps, 2020). Classical NETosis is driven by NADPH oxidase–dependent production of reactive oxygen species. This process triggers chromatin decondensation through activation of protein-arginine deiminase 4 (D’Cruz et al., 2018; Sollberger et al., 2018; Figure 2).

**FIGURE 2:**
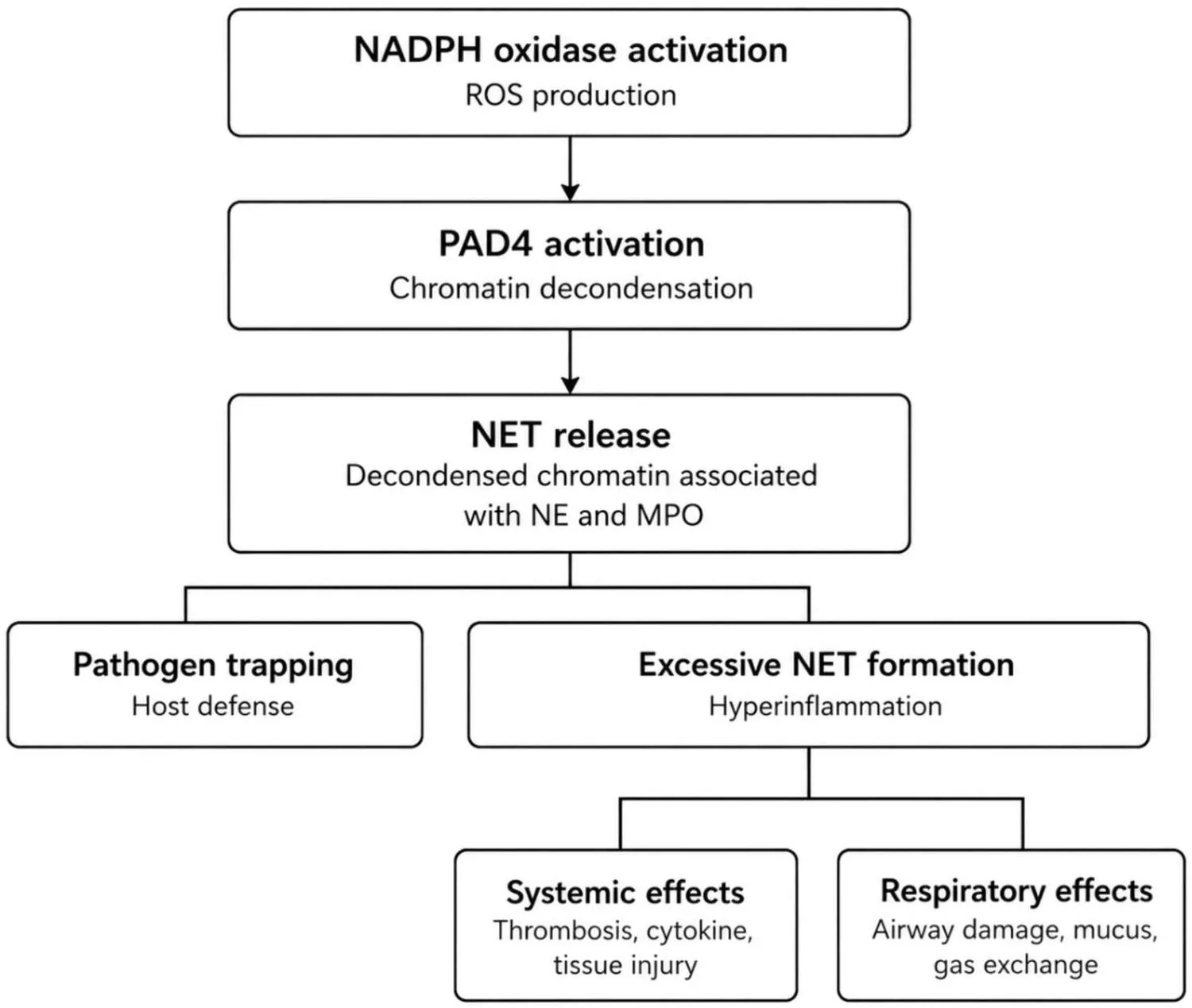
NETosis and tissue injury. ROS, reactive oxygen species; PAD4, protein-arginine deiminase 4; MPO, myeloperoxidase; NE, neutrophil elastase.

Although this mechanism contributes to host defense, uncontrolled NET formation plays a significant role in hyperinflammatory states (Fajgenbaum and June, 2020). During cytokine storms, recruited neutrophils release excessive NETs. This promotes thrombosis, amplifies cytokine production, and contributes to tissue injury (Fajgenbaum and June, 2020; Talasaz et al., 2022). In severe respiratory infections excessive NET formation damages the airway epithelium, enhances mucus production, and impairs gas exchange (Toussaint et al., 2017).

#### 3.3.3 Viral models of hyperinflammation

##### 3.3.3.1 Influenza

Influenza virus is a major cause of lung damage, which leads to severe disease due to dysregulated host inflammatory responses (Gu et al., 2021). Several influenza A subtypes have been associated with hyperinflammation (Taubenberger and Morens, 2006; De Jong et al., 2006). Respiratory epithelium infection triggers a cytokine cascade from epithelial cells and macrophages. In addition, highly pathogenic strains induce elevated levels of TNF-α which can lead to the development of acute respiratory distress syndrome (ARDS) (Lin et al., 2018; Short et al., 2014).

Cytokine storm can produce capillary thrombosis, focal necrosis and alveolar wall congestion. Furthermore, inflammatory infiltration, hyaline membrane formation, and pulmonary edema have also been described (Taubenberger and Morens, 2008). Host-related factors such as age, sex, and obesity may modulate the magnitude of the inflammatory response and influence patient’s prognosis (Gu et al., 2021).

TNF-α plays a central role in influenza inflammation by activating NF-κB and MAPK signaling pathways. It promotes leukocyte recruitment and amplifies cytokine production (Phung et al., 2011; Shi and Sun, 2018). Elevated TNF-α levels also damage the endothelial barrier integrity and disrupt tight junction proteins which can lead to capillary leakage, pulmonary edema, and lung injury (Ferreira et al., 2023; Pandey and Karupiah, 2022). In addition, NLRP3 inflammasome activation promotes the maturation of pro-inflammatory cytokines. This process requires a two-signal mechanism involving NF-κB–mediated priming followed by inflammasome assembly triggered by viral components and cellular stress signals (Ong et al., 2017).

##### 3.3.3.2 SARS-CoV-2 infection

SARS-CoV-2 infection is characterized by a broad spectrum of clinical manifestations from mild symptoms to life-threatening pneumonia associated with cytokine storm syndrome (Peiris et al., 2003; Ruscitti et al., 2022). In a subset of patients, dysregulated immune responses start 7–10 days after initial symptom onset. This may lead to clinical deterioration characterized by hyperinflammation, elevated levels of cytokines (IL-6, IL-10), and TNF-α, together with severe CD4+ and CD8+ T-cell lymphopenia and coagulopathy (Copaescu et al., 2020; Siddiqi and Mehra, 2020).

Interleukin-6 is a key mediator of the hyperinflammatory response in severe SARS-CoV-2 infection and a strong predictor of mortality (Zhou F. et al., 2020). The SARS-CoV-2 nucleocapsid protein can induce IL-6 expression through NF-κB activation, while elevated circulating levels are associated with inflammatory monocytes in critically ill patients (Zhang et al., 2007). High circulating IL-6 promotes acute-phase responses, endothelial dysfunction, vascular permeability, and pulmonary injury, contributing to ARDS and correlating with disease severity and respiratory failure (Channappanavar and Perlman, 2017; Herold et al., 2020).

Severe disease is also frequently associated with hyperferritinemia, which predicts intensive care admission, need for mechanical ventilation, and mortality (Ruscitti et al., 2022). Furthermore, SARS-CoV-2 further contributes to immune dysregulation through active evasion strategies: viral proteins such as ORF8 can inhibit interferon responses via epigenetic mechanisms, impairing antiviral immunity and facilitating disease progression (Mantovani and Garlanda, 2023).

##### 3.3.3.3 RSV infection

Hyperinflammation in RSV infection is characterized by an intense inflammatory response in the lower respiratory tract driven by several mediators. Early release of pro-inflammatory cytokines (IL-6, IL-1β, IL-10, CCL5, and TNF-α) by airway epithelial cells, macrophages, and dendritic cells contributes to disease severity (Bohmwald et al., 2019).

CXCL8 is responsible for neutrophil recruitment to the airways, where the release of reactive oxygen species and NETs leads to epithelial injury (Noah and Becker, 1993). RSV also skews adaptive immunity toward Th2 and Th17 responses, characterized by the release of cytokines IL-4, IL-5, IL-13, IL-17A, and IL-22. These responses promote eosinophilic inflammation, mucus hypersecretion, and airway remodeling in neonatal mice (Zhang et al., 2022). These processes are initiated by viral recognition through pattern recognition receptors such as TLR3, TLR7, and RIG-I, and activation of the NLRP3 inflammasome, while RSV simultaneously suppresses type I and III interferon responses through the viral proteins NS1 and NS2 (Spann et al., 2005).

Infants are especially susceptible due to immature Th2/Th17 immunity and reduced interferon production. In contrast, older adults are affected by immunosenescence and inflammaging, with impaired NK cell, dendritic cell and CD8 ^+^ T cell functions. These alterations reduce antibody affinity and memory B-cell responses while promote exaggerated inflammation and tissue damage, particularly in individuals with comorbidities (Georgakopoulou and Pitiriga, 2025; Shaw et al., 2013).

#### 3.3.4 Mechanisms of immune evasion

SARS-CoV2, Influenza and VRS have developed multiple strategies to evade innate immune responses. It should be noticed type I and III interferon signaling suppression, disrupting pattern recognition receptor pathways, and impairing antiviral cytokine production (Kikkert, 2020; Liu et al., 2024). These mechanisms differ across viral species.

In RSV, the non-structural proteins NS1 and NS2 are the main immune antagonists (Spann et al., 2005). These proteins inhibit IFN production and RIG-I and MDA-5 activation. They block IRF3 and STAT nuclear translocation, and promote the degradation of TRAF3 and STAT2 (Efstathiou et al., 2024).

SARS-CoV-2 targets IFN induction and signaling (Lei et al., 2020). NSP1 suppresses host mRNA translation and interferes with JAK/STAT pathways, while NSP13, ORF6, and the papain-like protease inhibit TBK1, IRF3, STING, and STAT signaling pathways (Kumar et al., 2021).

Influenza virus also suppresses innate antiviral responses, through NS1 protein (Wang et al., 2022). NS1 inhibits RIG-I signaling, sequesters double-stranded RNA to avoid PRR recognition, and blocks TRAF3-mediated IFN production (Wang et al., 2022).

### 3.4 Biomarkers of inflammation

Biomarkers of inflammation are relevant in the management of SARS-CoV-2, Influenza and VRS infections and their associated hyperinflammatory syndromes. Beyond their diagnostic value, they may help estimate disease severity, identify patients with dysregulated inflammatory responses who may benefit from immunomodulatory therapies, and support risk stratification in clinical practice. In addition, they can be useful for tracking disease progression and guiding treatment escalation. The most important biomarkers have been summarized in Table 2.

**TABLE 2:** Diagnostic and prognostic performance of interleukin-6 (IL-6), ferritin, and C-reactive protein in COVID-19, influenza and VRS.

Virus	Marker	AUC	Cutoff	Sensitivity/specificity	Evidence
COVID-19	IL-6	0.85 (severity); 0.53 (mortality)	20–87 pg/mL	65%–94%/68%–94%	Strong evidence from meta-analyses and multiple cohorts; best validated biomarker for disease severity (Liu et al., 2022; Santa Cruz et al., 2021).
	Ferritin	0.65–0.79	490–1,873 ng/mL	58%–92%/58%–96%	Moderate prognostic performance with considerable heterogeneity across studies (Lino et al., 2021).
	CRP	0.69–0.88	4.8–6.8 mg/dL	66%–76%/67%–90%	Well-validated; diagnostic performance similar but generally slightly inferior to IL-6 (De Rop et al., 2024).
Influenza	IL-6	Not consistently reported	No validated cutoff	No data available	Strong association with ICU admission and mortality, although validated ROC are lacking (Paquette et al., 2012).
	Ferritin	0.72 (poor clinical outcome)	≥500 ng/mL	No data available	Hyperferritinemia is associated with respiratory failure and poor outcomes (Lalueza et al., 2020).
	CRP	0.82 (ICU admission)	6.4 mg/dl	82%/68%	Consistently associated with severe disease but limited data (Zimmerman et al., 2010).
RSV	IL-6	0.85 (diagnostic and severity)	No validated cutoff	No data available	Associated with disease severity, single study (Iftimie et al., 2024; Lui et al., 2021).
	Ferritin	No data available	No data available	No data available	No prognostic evidence is currently available.
	CRP (children)	0.61–0.72 (severity); 0.87 (bacterial co-infection)	1.9–3.51 mg/dL	No data available	Limited value for predicting RSV severity; performs better in identifying bacterial co-infection in children (Adar et al., 2025).

AUC, area under curve, CRP, C reactive protein, ICU, intensive care unit.

#### 3.4.1 Ferritin

Ferritin plays a complex and dual role in viral infections. It acts as an essential intracellular iron-storage protein and as a mediator of inflammation. Viruses can specifically target and regulate proteins involved in iron homeostasis. Imbalances in iron metabolism in host cells caused by viral infection can induce inflammation (Schmidt, 2020). During infection, increased ferritin levels reflect a protective host response aimed at supporting an important host defense mechanism that deprives bacterial growth and protects immune cell function (Kernan and Carcillo, 2017).

However, iron dysregulation has been reported in critically ill COVID-19 patients and is associated with elevated lung iron levels, suggesting a link between iron metabolism and respiratory dysfunction (Duca et al., 2021). Beyond its role in iron storage, ferritin contributes to inflammation through the promotion of NF-κB signaling and IL-1β production, thereby amplifying the inflammatory response (Ruddell et al., 2009).

In hospitalized patients with Influenza A, hyperferritinemia is commonly defined by a threshold of 500 ng/mL. Levels exceeding this value are associated with a 5-fold increase in the odds of poor outcomes, including respiratory failure, intensive care unit (ICU) admission, and death (Lalueza et al., 2020). In very elderly patients (≥85 years), elevated ferritin has been identified as an independent predictor of in-hospital mortality following influenza infection (Arrieta et al., 2022).

Hyperferritinemia syndromes are characterized by extremely high ferritin levels. These are a group of life-threatening conditions that include macrophage activation syndrome, adult-onset Still’s disease, and hemophagocytic lymphohistiocytosis (Zhao X. et al., 2024). During viral infections, proinflammatory cytokines upregulate ferritin production. This response further amplifies systemic inflammation and contributes to the development of cytokine storm (Fajgenbaum and June, 2020; Kernan and Carcillo, 2017).

#### 3.4.2 C-reactive protein

C-reactive protein (CRP) is the most widely recognized acute phase protein in humans and a soluble molecule of the innate immune response. It is a member of the short pentraxin family, which is synthesized by hepatocytes in response to inflammatory triggers. CRP concentrations can increase up to 1,000-fold during acute infection (Mantovani and Garlanda, 2023). CRP also participates in host defense and recognizes pathogen associated molecular patterns and phospholipid exposed on damaged or apoptotic cells (Zhou et al., 2024). Complement system is activated by CRP and promotes opsonization, as well as the clearance of microorganisms and cellular debris.

In addition, CRP binds to Fcγ receptors on phagocytic cells. This action enhances the phagocytosis of pathogens and apoptotic cells and can help to the regulation of inflammatory responses (Lu et al., 2008). Owing to its rapid induction and close association with inflammatory activity, CRP is widely used in clinical practice as a sensitive marker of infection and systemic inflammation. However, accumulating evidence indicates that elevated CRP levels are more likely to reflect underlying inflammatory processes rather than direct role in disease pathogenesis (Zhou et al., 2024).

#### 3.4.3 Interleukin 6

Interleukin-6 is a cytokine produced in response to infection or tissue damage and is essential for the regulation of immunity (Tanaka et al., 2014). JAK/STAT3 pathway is activated by IL-6 which induces immune signaling, inflammation, and cellular responses (Wang et al., 2013). This cytokine promotes the differentiation of monocyte into macrophages and enhances B-cell IgG production. Additionally, it inhibits dendritic cell maturation and promotion of the Th2 response (Chomarat et al., 2000; Park et al., 2004; Yang et al., 2016). In addition, IL-6 is involved in the transition from innate to adaptive immunity by the differentiation of T follicular helper cells (Piattini et al., 2025). Moreover, it also promotes local complement production in the lungs, particularly C3 and C5 (Piattini et al., 2025).

The biological consequences of IL-6 have been associated with both pro- and anti-inflammatory effects (Scheller et al., 2011). Elevated systemic levels of IL-6 have been associated with severe clinical outcomes in viral infections, although it is controversial whether is beneficial or detrimental to host (Iftimie et al., 2024). Excessive or dysregulated IL-6 production may impair antiviral immunity. High IL-6 levels impair CD8^+^ T-cell function through reduce IFN-γ production and altered T-cell differentiation (Yang et al., 2016). They also enhance Th17 responses, which contribute to inflammation and immunopathology (Martinez et al., 2012). Furthermore, IL-6 has been linked to the upregulation of inhibitory pathways, which diminish cytotoxic T-cell activity and may favor viral persistence, ultimately compromising effective viral clearance (Bardhan et al., 2016).

During viral infections, two hypotheses have been proposed to explain the increase in IL-6. Firstly, highly virulent strains may evade immune responses more effectively, which results in higher viral loads and increase of IL-6. Secondly, polymorphisms in the IL-6 gene may cause excessive IL-6, which contributes to severe disease outcomes (Velazquez-Salinas et al., 2019).

In viral respiratory infections, this cytokine is a valuable biomarker. Elevated IL-6 is independently associated with severity in adults with influenza, COVID-19, and RSV infection. IL-6 shows strong diagnostic and prognostic performance, with areas under the curve exceeding 0.85, and a high ability to discriminate severe disease and adverse outcomes in COVID-19 (Liu et al., 2022; Santa Cruz et al., 2021).

#### 3.4.4 Interleukin 1 (IL-1)

Interleukin-1 is a key mediator of the inflammatory response (Bawazeer et al., 2021). While IL-1β release depends on viral entry and caspase-1 activity, IL-1α release is independent of caspase-1 (Piper et al., 2013). The recruitment and activation of immune cells is mediated by IL-1. This process is critical for viral clearance and survival (Schmitz et al., 2005).

The blockade of IL-1 has been described in rhinovirus infection. It reduces pro-inflammatory mediator production and neutrophil chemoattractant expression without impairing interferon activity. These findings suggest that IL-1 inhibition may attenuate harmful inflammation without compromising antiviral immunity (Piper et al., 2013). However, the potential benefit must be balanced against the risk of impairing antiviral immune responses (Bawazeer et al., 2021; Dinarello, 2011).

#### 3.4.5 Pentraxin 3

Pentraxin 3 (PTX3) is a soluble pattern recognition receptor that is a humoral component of the innate immune system. It is produced locally at sites of inflammation by endothelial cells, macrophages, and neutrophils (Balhara et al., 2013). PTX3 is relevant in respiratory diseases, contributing to pathogen recognition, complement activation, opsonization, and early clearance.

Pentraxin 3 levels increase early and correlate with disease severity in ARS. This reflects the activation of innate immunity and neutrophil driven inflammation. Experimental models have shown that PTX3 deficiency is associated with increased susceptibility to lung injury, whereas exogenous PTX3 may exert protective effects by modulating inflammatory responses (Han et al., 2011, 2012).

In Influenza, PTX3 interacts with viral components. It binds to hemagglutinin and inhibits viral attachment to epithelial cells, while also interferes with neuraminidase activity and limits the release of newly formed viral particles (Reading et al., 2008).

#### 3.4.6 Apolipoprotein H

Apolipoprotein H (ApoH) or Beta-2 Glycoprotein 1, is a plasma protein related to innate immunity. It regulates inflammatory and thrombotic responses during viral infections (Garcinuño et al., 2024; Yu Z. et al., 2024).

Apolipoprotein H acts as a regulator of complement activation and coagulation pathways. This protein maintains immune balance and prevents excessive inflammatory responses that could lead to tissue damage or organ dysfunction (McDonnell et al., 2020). During infection, ApoH can also act as an opsonin by binding to viral particles and microvesicles which contain phosphatidylserine, thereby facilitating their clearance.

Evidence from several viral infections suggests that ApoH plays a protective role. In patients hospitalized with influenza, plasma ApoH levels are significantly lower than those observed in healthy individuals. Low ApoH concentrations have reported as an independent risk factor for respiratory failure (Garcinuño et al., 2024). This deficiency may sustain a pro-inflammatory state, which contributes to tissue damage and disease progression. Finally, reduced ApoH levels compared with healthy controls have been also reported in pediatric patients with sepsis (Yu Z. et al., 2024).

### 3.5 Immunomodulation in severe viral respiratory infection

#### 3.5.1 The ideal immunomodulator

Given the immune alterations associated with viral infections, there is a clear need to define optimal immunomodulatory strategies. An ideal approach should have a well-balanced immunological profile.

First, it should selectively modulate pathological inflammation without inhibiting the initial antiviral immune response (Mulik et al., 2023). The intervention should also have a rapid onset of action and a controlled duration, allowing restoration of effective viral clearance once the hyperinflammatory phase has been contained. Safety is essential, with a low risk of secondary infections or viral reactivation. The ideal immunomodulator should target key pro-inflammatory mediators such as IL-6, IL-1, and TNF-α, or block pathological signaling pathways such as JAK–STAT and NLRP3 inflammasome activation, while preserving type I and type III interferon responses (Liu et al., 2016; Figure 3).

**FIGURE 3:**
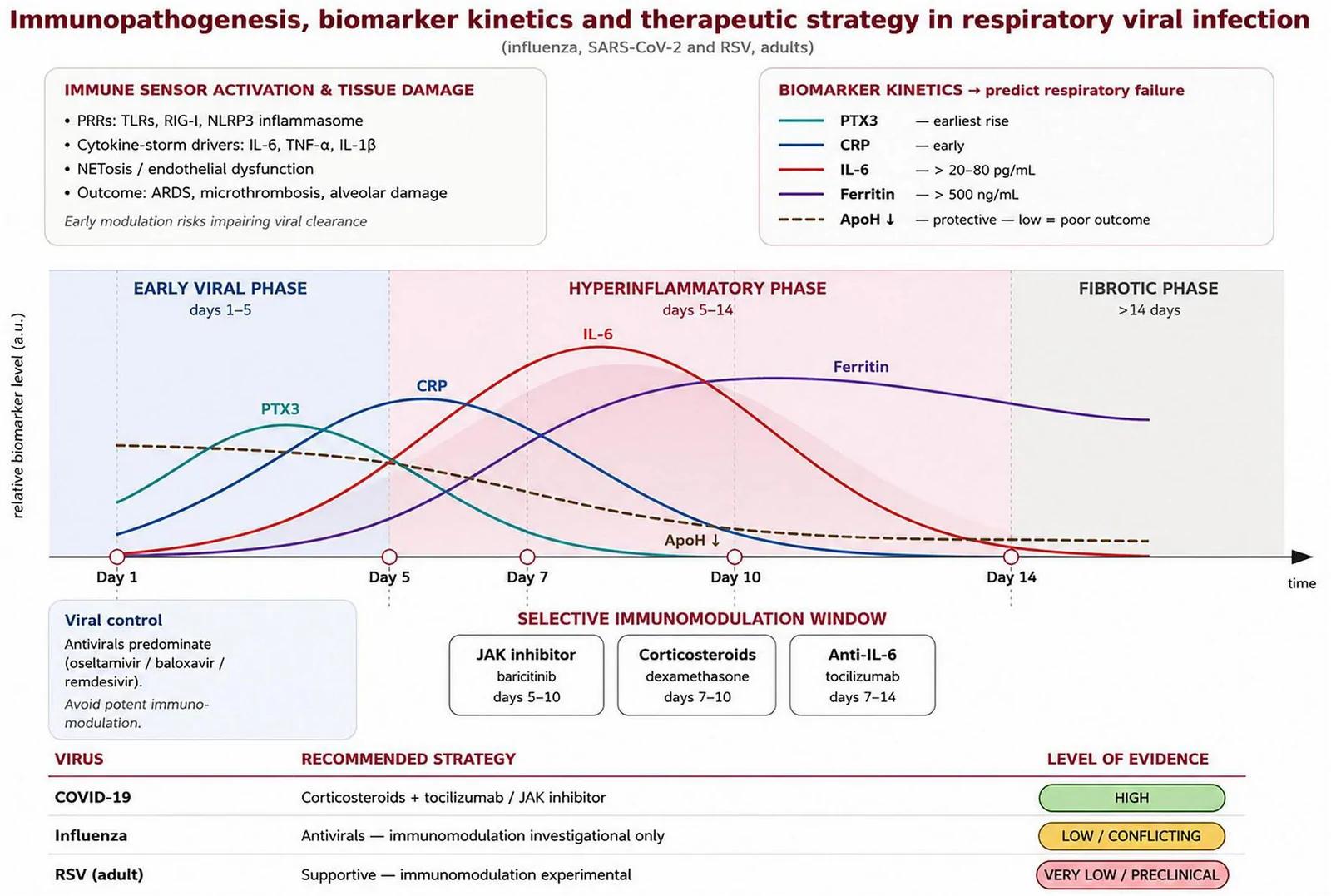
Immunopathogenesis, biomarker kinetics and therapeutic strategy in respiratory viral infection.

In addition, it should promote the resolution of inflammation by enhancing regulatory mediators or immune cells that limit tissue damage. Finally, pharmacological characteristics are also important: the therapeutic agent should achieve rapid bioavailability in the lung or reticuloendothelial system and have a limited duration of action in order to minimize the risk of prolonged immunosuppression.

#### 3.5.2 Timing of intervention

The timing of immunomodulatory therapy during viral infections is a critical determinant of clinical outcomes. Two main phases can be distinguished based on the underlying pathophysiology. Different intervention moments are shown in Figure 3. Moreover, signaling pathways and therapeutic targets have been illustrated in Figure 4.

**FIGURE 4:**
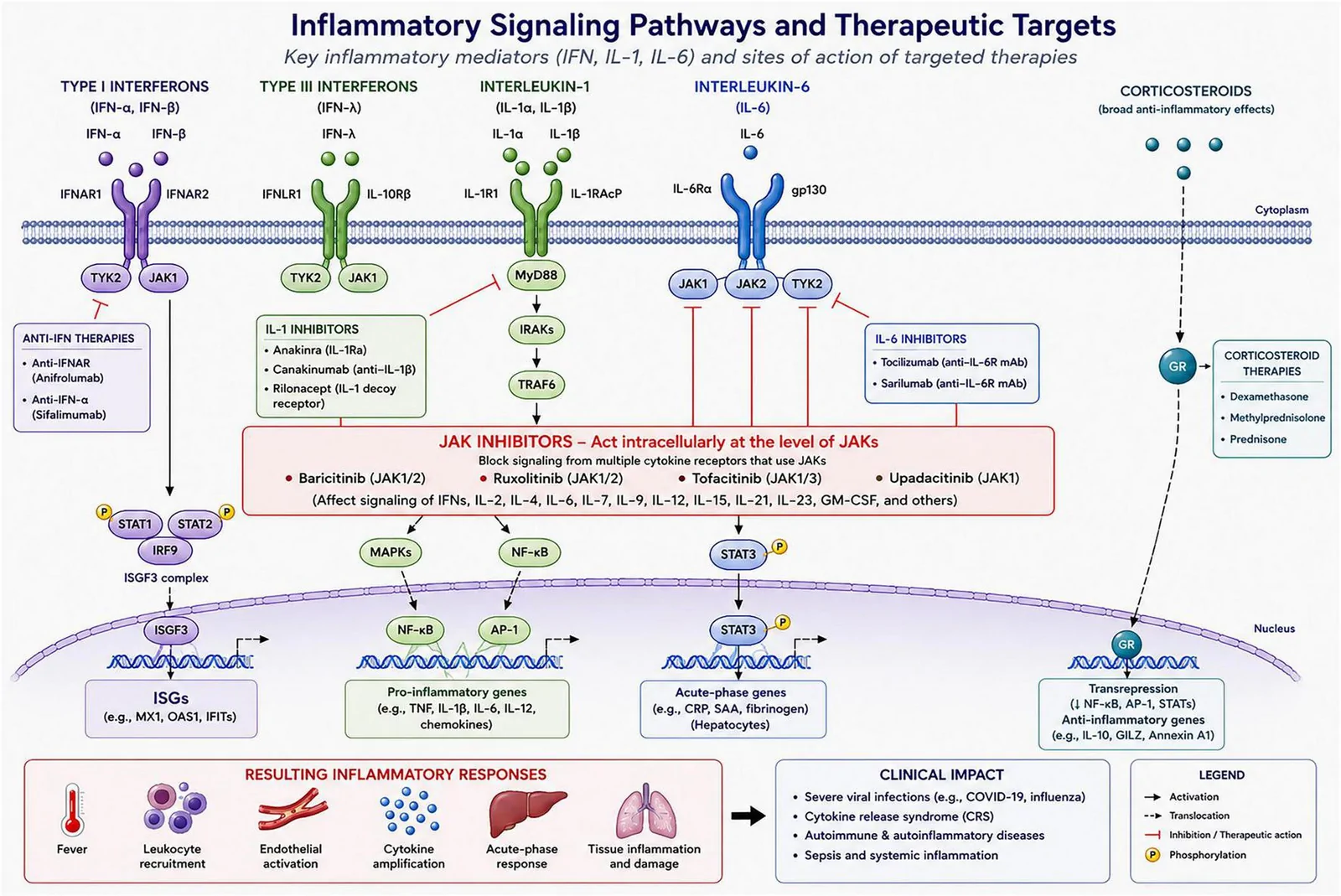
Inflammatory signaling pathways and therapeutic targets: interferons, IL-1, IL-6, JAK inhibitors and corticosteroids.

##### 3.5.2.1 Early viral phase

During the initial stage of infection, typically within the first 1–5 days after symptom onset, viral replication predominates and the primary therapeutic objective is viral control. At this stage, early immunomodulation may attenuate the antiviral immune response and potentially prolong infection (Lee et al., 1999). For this reason, potent immunomodulatory strategies are generally not recommended during the early phase (Chiaretti et al., 2013; Choy et al., 2020), except in highly selected cases, such as the presence of an early hyperinflammatory syndrome.

##### 3.5.2.2 Inflammatory or post-replicative phase

From the fifth day after the onset of symptoms until days 10–14, host-mediated inflammatory injury becomes the predominant driver of disease. This phase is characterized by progressive hypoxemia and radiological infiltrates. Laboratory findings often include elevated IL-6 levels and hyperferritinemia. This moment represents the most appropriate window for immunomodulatory intervention (Choy et al., 2020). Late inhibition may improve outcomes due to the attenuation of excessive inflammation (Choy et al., 2020; Kishimoto, 2005). Selective immunomodulators guided by biomarker profiles should be used to reduce pulmonary inflammation while preserving essential antimicrobial defense mechanisms.

According to the respiratory virus, the transition from viral replication to host inflammatory is dynamic and may vary. It also depends on host characteristics, immune status, and disease severity. The optimal timing of immunomodulation should be based on the integrated assessment of the clinical course, respiratory deterioration and oxygen requirements.

However, immunomodulation may increase the risk of secondary infections (Gioia et al., 2024; Hui and Chan, 2025). Therefore, careful clinical monitoring, the use of the minimal effective dose, and prophylaxis in high-risk patients should be considered. Corticosteroids, cytokine blockade, JAK inhibitors, and interferon modulation evidence has been summarized in Table 3 to facilitate the distinction between established and investigational approaches across COVID-19, influenza, and RSV. Clinical use of immunomodulators, pharmacokinetic characteristics and dose adjustment has been included in Table 4.

**TABLE 3:** Current level of evidence of host-directed immunomodulatory therapies in COVID-19, influenza, and respiratory syncytial virus (RSV).

Viral infection	Drug/intervention	Level of evidence	Clinical status
COVID-19	Corticosteroids	High – randomized clinical trials and meta-analyses (The Recovery Collaborative Group, 2021)	Established clinical use in selected hospitalized patients requiring oxygen or respiratory support
	Tocilizumab (IL-6 inhibitor)	High – randomized clinical trials and meta-analyses (Abani et al., 2021; Salama et al., 2021)	Recommended in selected patients with severe or critical disease and systemic inflammation
	Sarilumab (IL-6 inhibitor)	Moderate/high – clinical trials and meta-analysis (REMAP-CAP Investigators, 2021)	Recommended in critically ill patients
	Siltuximab (IL-6 inhibitor)	Low- heterogeneous data (Gritti et al., 2020)	Investigational; research settings only
	IL-1 blockade	Moderate/limited – clinical trials with heterogeneous results (Davidson et al., 2022)	Not routinely recommended; potential role in selected phenotypes
	JAK inhibitors	Moderate – randomized clinical trials and meta-analyses but heterogeneous (Recovery Collaborative Group, 2022)	Recommended in selected hospitalized patients with severe disease
	Interferon modulation	Moderate (Kalil et al., 2021; Ryoo et al., 2023)	Not recommended
Influenza	Corticosteroids	Low/conflicting – predominantly observational evidence (Lansbury et al., 2020)	Not recommended for routine treatment of influenza
	IL-6 blockade	Low, experimental trials (Pandey et al., 2023)	Investigational; research settings only
	IL-1 blockade	Low – limited clinical and preclinical evidence (Bawazeer et al., 2021)	Investigational; research settings only
	JAK inhibitors	Low – limited clinical and preclinical evidence (Yu Y. et al., 2024)	Investigational; research settings only
	Interferon modulation	Preclinical (Sohail et al., 2022; Barman et al., 2021)	Experimental; research settings only
RSV	Corticosteroids	Low – clinical studies with limited or context-dependent benefit (Fernandes et al., 2013; Plint et al., 2009; Lee et al., 2011)	Not routinely recommended for RSV infection
	IL-1 blockade	Preclinical/limited clinical evidence (Roberts et al., 1986; Schuler et al., 2020)	Investigational; research settings only
	JAK inhibitors	Preclinical/limited clinical evidence	Investigational; research settings only
	Interferon modulation	Preclinical in adults/low evidence in children (Chen et al., 2020; Chipps et al., 1993)	Experimental; research settings only

**TABLE 4:** Clinical use of immunomodulators in respiratory viral infections, pharmacokinetic characteristics and dose adjustment.

Drug		Dosage*	Key PK/PD	Renal and hepatic adjustment	Infection-related safety	Regulatory status	FDA boxed warning	References
Corticosteroids (dexamethasone)		6 mg PO or IV once daily for up to 10 days (RECOVERY trial). No evidence supporting dexamethasone over other corticosteroids. No established therapeutic regimen for influenza or RSV.	- Tmax :1 h. - Protein binding: 77%. - Terminal t½: 4 h. - Renal clearance: 15.7 L/h following a single dose. Less than 10% of total clearance is renal. The biological effect persists longer than the plasma half-life.	No specific evidence-based dose adjustment has been established in COVID-19 infection; clinical monitoring is advised.	Increased risk of bacterial, viral, fungal, parasitic and opportunistic infections, reactivate latent infections and mask signs of infection. Risk increases with dose and duration.	EMA-endorsed treatment option for hospitalized adults with COVID-19 requiring supplemental oxygen therapy o ventilatory support. No specific FDA-labeled COVID-19 indication.	None	Recovery Collaborative Group, 2022; European Medicines Agency, 2020; DailyMed, 2026a
IL-1 inhibitors	Anakinra	100 mg SC once daily for 7–10 days.	- SC bioavailability: 95%. - Tmax: 3–7 h - Terminal t½: 4–6 h. - Clearance increases with creatinine clearance and body weight.	Clearance is reduced by approximately 16%, 50%, 70% and 75% in mild, moderate, severe and end-stage renal impairment, respectively. For CrCl < 30 mL/min or end-stage renal disease, consider administration of the prescribed dose every other day. No formal hepatic pharmacokinetics studies; caution in severe hepatic impairment.**	In RA trials serious infections occurred in approximately 2% versus <1% with placebo (not a COVID-19-specific estimates). Avoid initiation during an active infection.	EMA-authorized treatment for adults with COVID-19 and pneumonia requiring supplemental oxygen who are at risk of developing severe respiratory failure (protein suPAR ≥ 6 ng/mL). FDA-EUA for a similar adult population.	None	DailyMed, 2025a; US Food and Drug Administration, 2026a,b; European Medicines Agency, 2025a
	Canakinumab	450 mg (40–60 kg); 600 mg (60–80 kg); 750 mg (>80 kg) IV once (investigational CAN-COVID regimen)	Available regulatory PK data mainly derive from approved SC indications rather than the IV COVID-19 regimen.** After 150 mg SC: - Cmax: 16 ± 3.5 μg/mL. - Bioavailability: 66%. - Terminal t½: 26 days. Pharmacokinetics is body-weight dependent.	No formal renal or hepatic pharmacokinetics studies.**	Increased risk of serious infections, specially of the upper respiratory tract. Avoid use with active, serious or opportunistic infection.**	No FDA/EMA approval for COVID-19, influenza or RSV.	None	Caricchio et al., 2021; DailyMed., 2026b; European Medicines Agency, 2017
JAK inhibitors (Baricitinib)		4 mg PO once daily for 14 days or until hospital discharge. Enteral administration is possible.	- Oral bioavailability: 80%. - Tmax: 1 h. - Protein binding: 50%. - Terminal t½: 10.8 h in intubated patients with COVID-19. - Renal clearance: 75%. Body weight does not require a separate adjustment.	Dosage modification for patients with COVID-19 and renal impairment is needed: eGFR 60– <90: 4 mg/day; 30– <60: 2 mg/day; 15– <30: 1 mg/day; <15, dialysis, ESRD or acute kidney injury: not recommended. Not known if dosage adjustment is needed in severe hepatic impairment, use only when potential benefit outweighs risk.	In COVID-19 trials, overall infections 14.8% vs. 16.7%, serious 7.5% vs. 9.2%, opportunistic 0.9% vs. 1.1% with baricitinib vs. placebo. Avoid use with active, serious or opportunistic infection.	FDA-approved for hospitalized adults with COVID-19 requiring supplemental oxygen, non-invasive or invasive ventilation, or ECMO. No EMA-authorized.	Serious infections, mortality, malignancy, major adverse cardiovascular events and thrombosis.	DailyMed., 2026c; European Medicines Agency, 2023
IL-6 antagonists	Tocilizumab	8 mg/kg IV over 60 min, maximum 800 mg. If worsen or do not improve, one additional infusion may be administered at least 8 h after the initial infusion.	- Cmax: 151 mcg/mL for one dose. - Eliminated by a combination of lineal clearance and non-linear elimination. No separate ICU or obesity adjustment is recommended.	No adjustment in mild or moderate renal impairment. Severe renal impairment and hepatic impairment has not been formally studied. In COVID-19, initiation is not recommended when AST or ALT is >10× ULN.	Increased risk of active tuberculosis, invasive fungal, bacterial, viral and other infections due to opportunistic pathogens.**	FDA and EMA-approved for hospitalized adults with COVID-19 receiving corticosteroids and requiring supplemental oxygen, non-invasive or invasive ventilation, or ECMO. FDA-approved label includes pediatric patients ≥ 2 years of age.	Risk of serious infections	REMAP-CAP Investigators, 2021; DailyMed., 2025b; European Medicines Agency, 2021
	Sarilumab	400 mg IV once (investigational REMAP-CAP regimen)	Pharmacokinetics of the single 400-mg IV COVID-19 regimen has not been adequately characterized. With the approved 200 mg SC every-2-weeks regimen in RA: - Cmax: 35.6 ± 15.2 mg/L. - Terminal t½: up to 10 days. No separate ICU or obesity adjustment is recommended.**	No adjustment in mild or moderate renal impairment. Severe renal impairment and hepatic impairment has not been formally studied. Treatment is not recommended in active hepatic disease.**	Increased risk of active tuberculosis, invasive fungal, bacterial, viral and other infections due to opportunistic pathogens.** REMAP-CAP did not identify an overall increase in serious adverse events.	No FDA/EMA approval for COVID-19, influenza or RSV.	Risk of serious infections	DailyMed., 2026d; European Medicines Agency, 2025b
	Siltuximab	11 mg/kg IV once (investigational COV-AID regimen)	With the approved 11-mg/kg IV every-3-weeks regimen: - Cmax: 332 μg/mL. - Terminal t½: 20.6 days.** Body weight was the main PK covariate. COVID-19 single-dose and ICU-specific PK data remain limited.	No adjustment for CrCl ≥ 15 mL/min or mild–moderate hepatic impairment.**	Avoid administration during a severe active infection because of IL-6 neutralization (may suppress fever and CRP).**	No FDA/EMA approval for COVID-19, influenza or RSV.	None	Declercq et al., 2021; DailyMed, 2024; European Medicines Agency, 2018
Interferons: heterogeneous formulations		No single class-wide regimen can be defined. ACTT-3 regimen is based in 4 doses of either 44 μg interferon beta-1a administered with remdesivir. The TOGETHER trial protocol involves the administration of 180 μg of pegylated interferon lambda Other studies used different interferon subtypes, doses and routes.	PK/PD varies substantially between interferon alfa, beta and lambda formulations, as well as between conventional and pegylated products.	Product and formulation specific. No class-wide renal or hepatic adjustment can be provided.	No valid class-wide estimate of secondary-infection risk. Safety findings depend on the interferon subtype, route, timing and severity of illness.	No FDA/EMA approval for COVID-19, influenza or RSV.	No applicable class-wide.	US Food and Drug Administration, 2026b; European Medicines Agency, 2019; Kalil et al., 2021; Reis et al., 2023

*Unless otherwise specified, the dosing regimens shown refer to COVID-19. No established immunomodulatory dosing regimen is available for adult influenza or RSV infection.

**Several PK values, renal and hepatic adjustment and risk of infection derive from the approved subcutaneous formulations and non-COVID-19 indications and should not be interpreted as PK estimates for the investigational intravenous COVID-19 regimens except if it is specified. RA, rheumatoid arthritis; PK, pharmacokinetics; PD, pharmacodinamics; Tmax, time to maximum concentration; Cmax, maximum concentration; FDA, Food and Drug Administration; EMA, European Medicines Agency.

#### 3.5.3 Broad immunomodulators: corticosteroids

Corticosteroids are broad-spectrum anti-inflammatory agents that have traditionally been used in infectious diseases due to their immunosuppressive effects (Zoorob and Cender, 1998). However, they exert potent immunomodulation. These drugs suppress IL-1β, TNF-α, and IL-6 and promote IL-10 (Brattsand and Linden, 1996). Despite these effects, corticosteroids have demonstrated clinical benefit in specific infectious situations.

In septic shock, corticosteroids have been associated with reduced organ dysfunction and shorter duration of vasopressor support. However, mortality benefits remain inconsistent across trials. In 2002, one of the first studies suggested that corticosteroids reduced 28-day mortality (Annane et al., 2002). This finding was not confirmed by the CORTICUS trial in 2008, though hydrocortisone was associated with faster shock resolution (Sprung et al., 2008). In the Annane et al. (2002), corticosteroids significantly reduced mortality and increased vasopressor-free days and shorter duration of mechanical ventilation (Annane et al., 2018). In contrast, the Venkatesh et al. (2018) showed faster shock resolution and earlier ICU discharge, but no mortality benefit (Venkatesh et al., 2018).

Similarly, corticosteroids have shown benefit in certain severe respiratory infections The CAPE COD trial demonstrated that early corticosteroid therapy reduced mortality in patients with community-acquired pneumonia (Dequin et al., 2023; Venkatesh et al., 2018). Likewise, the RECOVERY trial showed a benefit in patients who received invasive mechanical ventilation or oxygen in COVID-19 (The Recovery Collaborative Group, 2021; Wagner et al., 2022).

However, the use of corticosteroids in influenza remains controversial. Some studies suggest potential benefits depending on patient characteristics and disease severity (Martin-Loeches and Torres, 2021; Ramos-Rincón et al., 2025). Moreover, a large prospective observational study reported reduced mortality in patients with severe hypoxemia treated with low-to-moderate corticosteroid doses (Zhou Y. et al., 2020). In contrast, large meta-analyses reported an increased mortality, higher rates of nosocomial infections, and no reduction in duration of mechanical ventilation or ICU stay (Lansbury et al., 2020; Ni et al., 2019; Zhou Y. et al., 2020).

In contrast, in RSV infection, most evidence regarding corticosteroid therapy derives from pediatric populations with viral bronchiolitis. Current evidence does not support a clinically relevant effect of systemic or inhaled glucocorticoids on admissions or length of hospitalization in infants and young children (Fernandes et al., 2013). A multicenter study showed that combined treatment with epinephrine and dexamethasone reduced hospital admissions and shortened symptom duration, although no benefit was observed when either treatment was used alone (Plint et al., 2009).

Corticosteroid use in RSV infection in adults is scarce. No clinical trials have evaluated the effect of corticosteroids in hospitalized adults with RSV infection (Pirracchio et al., 2024). Observational studies suggest that short courses of systemic steroids in patients hospitalized with RSV infection do not affect viral load or shedding (Lee et al., 2011). Moreover, in another observational study of patients with allogeneic stem cell transplant recipient corticosteroid use did not improve RSV-related outcomes including long-term pulmonary function (Damlaj et al., 2016).

#### 3.5.4 Targeted cytokine blockade

##### 3.5.4.1 IL-6 pathway

Targeting the IL-6 signaling pathway represents a promising strategy for the treatment of hyperinflammation in severe viral infections.

###### 3.5.4.1.1 Tocilizumab

Tocilizumab is a recombinant humanized monoclonal antibody targeting the soluble and membrane-bound IL-6 receptor (Choy et al., 2020). The REMAP-CAP and RECOVERY trials showed that, in hospitalized patients with COVID-19 pneumonia who did not require invasive mechanical ventilation, tocilizumab reduced progression to mechanical ventilation or death (Abani et al., 2021; Salama et al., 2021; Agarwal et al., 2020). Patients with early signs of hyperinflammation and limited organ damage may benefit from IL-6 pathway. On the other hand, those treated later in the course of disease may experience a limited clinical benefit (Potere et al., 2021).

Targeting IL-6 signaling may also reduce hyperinflammation in severe influenza; however, may impair innate immunity (Kang and Kishimoto, 2021). The main challenge lies in balancing the reduction of harmful inflammation while preserving the essential antiviral functions of IL-6 (Li X. et al., 2025). Experimental trials evaluating IL-6 inhibitors, have shown mixed results (Pandey et al., 2023).

###### 3.5.4.1.2 Sarilumab

Sarilumab is a fully human monoclonal antibody that antagonizes soluble and membrane-bound IL-6 receptors (Choy et al., 2020). Although early studies suggested potential benefits, most randomized clinical trials do not demonstrate a significant improvement in clinical outcomes (CORIMUNO-19 Collaborative Group, 2022; Lescure et al., 2021). However, further meta-analyses have suggested a benefit in critically ill patients, particularly when sarilumab is administered with corticosteroids (REMAP-CAP Investigators, 2021).

###### 3.5.4.1.3 Siltuximab

Siltuximab is a chimeric monoclonal antibody that neutralizes IL-6 by preventing its binding to IL-6 receptors (Choy et al., 2020). Although siltuximab may constitute a potential therapeutic option in viral infections, evidence for its use in COVID-19 is limited and mainly based on observational data (Gritti et al., 2020). Few clinical trials addressing this indication have been published, and the available evidence remains heterogeneous and insufficient to establish a definitive clinical benefit (EusaPharma (UK) Limited, 2021).

As previously noted, IL-6 overexpression has been linked to more severe RSV infection, particularly in infants with prolonged hospitalization and increased oxygen supplementation needs (Becker et al., 1991). Nonetheless, no clinical evidence supports the use of IL-6 blockade for RSV infection in humans and no randomized trials or clinical practice guidelines recommend IL-6 inhibitors in this setting (McElvaney et al., 2021; Vázquez et al., 2019).

##### 3.5.4.2 IL-1 inhibitors

The NLRP3 inflammasome plays a central role in the pathogenesis of severe influenza by promoting IL-1β production, thereby driving hyperinflammation (Ong et al., 2017; Xu and Núñez, 2023). Consequently, IL-1 inhibition has been proposed as a therapeutic strategy, particularly in severe hyperinflammatory syndromes (Cavalli et al., 2016). Canakinumab neutralizes IL-1β, whereas anakinra blocks the IL-1 receptor (Cavalli and Dinarello, 2019; Chakraborty et al., 2012).

Available evidence in COVID-19 does not demonstrate clear benefit of IL-1 blockade in a Cochrane systematic review (Davidson et al., 2022). Similarly, therapeutic inhibition of IL-1β reduced airway inflammation and inflammatory cell infiltration just in murine model (Bawazeer et al., 2021).

In RSV infection, preclinical studies in animal models suggest that pharmacological IL-1 blockade may attenuate RSV-induced immunopathology and reduce asthma development; however, these findings have not yet been translated into human clinical trials (Roberts et al., 1986; Schuler et al., 2020).

##### 3.5.4.3 JAK inhibitors

Janus kinase (JAK) inhibitors block signaling mediated by the JAK family (JAK1, JAK2, JAK3, TYK2), which are essential for the transduction of signals from cytokines. These kinases regulate the JAK-STAT pathway, a key mediator of immune responses, inflammation, and antiviral defense (Ezeonwumelu et al., 2021).

In hospitalized patients with COVID-19, baricitinib reduced mortality, although the magnitude of this benefit was modest (Recovery Collaborative Group, 2022). Consistent with these findings, two systematic reviews and meta-analysis of clinical trials found that JAK inhibitors reduced death rate in all subgroups, regardless of the level of respiratory support or concomitant therapies. Moreover, it also may reduce the incidence of serious adverse events (Amstutz et al., 2025; Kow et al., 2024). However, a Cochrane review concluded that JAK inhibitors have little effect on overall clinical improvement (Kramer et al., 2022).

Evidence for JAK inhibitors in influenza is limited to preclinical studies. There is no clinical trial or guideline which supports their use in humans for influenza (Wang et al., 2020). In animal models, these agents reduce pro-inflammatory cytokine production JAK inhibitors decrease neutrophil and macrophage infiltration, and mitigate lung injury (Yu Y. et al., 2024). In addition, a preclinical *in vitro* study of tyrosine kinase inhibitors, has shown promising results in influenza (Meineke et al., 2022).

#### 3.5.5 Therapeutic interferon modulation

As it has been described, although IFNs are essential for viral clearance, excessive production may lead to T cell suppression, inhibition of lymphocyte development, and impaired tissue repair (Jung et al., 2023; McNab et al., 2015).

Type I interferons have been used in hepatitis B and C, where they exert both antiviral and immunomodulatory effects (Lin and Young, 2014). However, their use is limited by significant side effects as well as suboptimal response rates in some patients (Tang et al., 2018).

In experimental mouse models of severe influenza, higher levels of IFN-αβ were associated with increased lung injury, morbidity, and mortality (Davidson et al., 2014), whereas reduced IFN signaling limited tissue damage and improved outcomes. These findings highlight that an exaggerated antiviral response may contribute to disease severity (Davidson et al., 2014).

Several preclinical strategies targeting interferon-driven inflammation in influenza have shown beneficial effects. TLR7 antagonism reduces type I IFN and pro-inflammatory cytokines by modulating dendritic cells and monocytes while preserving epithelial antiviral responses, leading to decreased inflammation and disease severity (Rappe et al., 2021). Itaconate derivatives similarly suppress IFN responses and inflammation, reducing lung damage and mortality without increasing viral replication (Sohail et al., 2022). Sequential targeting of IFN pathways (type I IFN early and type II IFN later) further minimizes lung injury and improves outcomes in experimental models (Barman et al., 2021). In contrast, type III IFNs provide antiviral protection with limited inflammatory effects, as they primarily acts on epithelial cells without activating immune cells (Davidson et al., 2016).

In COVID-19, IFN therapy demonstrates a strong timing-dependent effect. Early administration in outpatients, may reduce hospitalization and mortality, especially when given within the first days of symptom onset and in patients with high viral loads (Reis et al., 2023). However, large clinical trials and meta-analysis in hospitalized patients with moderate-to-severe disease have not demonstrated significant benefits in terms of mortality, need for mechanical ventilation, or recovery time (Kalil et al., 2021; Ryoo et al., 2023; WHO Solidarity Trial Consortium, 2021).

Interferon therapy has shown modest clinical benefit in infant RSV. Nebulized IFN-α1b may improve respiratory symptoms, while intramuscular interferon has demonstrated limited efficacy (Chen et al., 2020; Chipps et al., 1993). No clinical data on the use of interferons in adult patients with RSV infection have been published, and the evidence is limited to preclinical studies (Higgins et al., 1990).

Preclinical studies support the potential role of interferons in RSV infection. Administration of IFN-α prior to infection reduces immunopathology, while intranasal IFN-α enhances mucosal immunity by increasing RSV-specific IgA production and inducing BAFF expression (Hijano et al., 2018, 2019). Despite these findings, IFN-α1b is not currently used in routine clinical practice for RSV. The available evidence remains limited by small clinical studies and heterogeneous routes of administration (Hijano et al., 2019).

## 4 Discussion

This review highlights that the clinical severity of SARS-CoV-2, Influenza and RSV is not only determined by viral replication. It also depends on the host early innate immune response. While early antiviral mechanisms are essential for pathogen clearance, hyperinflammation is determinant in lung injury, respiratory failure, and adverse outcomes.

A central concept from this work is the balance between effective antiviral defense and the risk of immunopathology. Pro-inflammatory cytokine production and interferon signaling, play a critical role during the early phase of infection. However, these responses may be amplified and persist over time and contribute to tissue damage. This can lead to cytokine storm, inflammasome activation, and NETosis. This dual nature is exemplified by mediators such as IL-6, IL-1, TNF-α, and type I interferons, which are essential for host defense under controlled conditions but may become harmful when their regulation is lost.

The identification of reliable biomarkers represents a critical step toward improving risk stratification and guiding therapeutic interventions. Among these, ferritin and IL-6 have emerged as indicators of hyperinflammation and disease severity. Ferritin is a potential mediator of inflammation, that self-amplifies inflammatory loop. Similarly, IL-6 reflects both immune activation and disease progression, although its dual role complicates its interpretation as a purely pathogenic factor. New molecules like ApoH have also been related to disease severity and immune dysregulation. Moreover, PTX3 is a new biomarker which is appropriated only for research settings and further studies are needed.

As it has been discussed, these findings suggest that broad immunosuppression is unlikely to be the optimal approach. Instead, strategies should focus on selectively modulate harmful inflammation and preserve antiviral immunity. This concept is supported by the heterogeneous results observed with different immunomodulatory agents. Corticosteroids have shown benefit in selected conditions such as severe COVID-19 and septic shock but have shown inconsistent or potentially harmful effects in influenza and limited evidence in RSV. Similarly, targeted therapies against IL-6, IL-1, and JAK–STAT pathways have shown variable efficacy, often influenced by patient selection and timing of administration. Treatments during the early viral phase may impair antiviral responses, whereas treatment during the hyperinflammatory phase may stop tissue damage and improve outcomes. However, when it is administered too late, once organ damage has become established, they may have no effect.

The integration of host-related factors, biomarkers of inflammation, and disease severity may contribute to improved risk stratification and therapeutic decision-making. In COVID-19 there is enough evidence which supports the benefit from immunomodulatory therapies (corticosteroids or tocilizumab). In influenza, IL-6 and ferritin have shown prognostic value, but there is not a certain role guiding immunomodulatory treatment remains uncertain. Finally, in adult RSV infection, evidence is even more limited, and there is a lack in biomarkers or specific immunomodulatory strategies. Further studies are needed to define the role of biomarkers in patient stratification and to identify which patients are most likely to benefit from immunomodulatory therapies.

This review has several limitations. First, the available evidence is highly heterogeneous. It includes different study designs, patient populations, disease severity, therapeutic strategies, and outcome definitions, which limit direct comparisons across studies. Most evidence comes from observational studies or preclinical models. In particular, infectious complications have been poorly characterized in the available studies. Second, most of the published data derive from patients with COVID-19, whereas evidence of these immunomodulatory therapies in influenza or RSV, remains scarce. Third, this work was not conducted as a formal systematic review. Although there was a search strategy, no formal systematic review methodology, risk of bias assessment or quantitative evidence synthesis was performed. Consequently, the possibility of selection bias and incomplete identification of relevant studies cannot be excluded.

Different questions have not been answered yet. First, reliable biomarkers are required to identify patients who may benefit from immunomodulatory therapy. These molecules should detect early hyperinflammation and been affordable for clinical use. Once these patients are identified, treatment should target specific pathways while preserving antiviral response and control inflammation. Finally, although respiratory viral infections have similarities, specific clinical trial should be designed to strengthen the evidence.

In conclusion, an excessive inflammatory response to respiratory viral infections can be harmful, leading to respiratory failure, organ dysfunction and even death. Early detection of this response using various biomarkers is essential for optimizing patient care. Finally, it is essential to obtain further evidence on the role of immunomodulatory drugs in controlling this inflammatory response without compromising the control of the infection itself, as well as on the complications associated with these drugs.
